# Prevalence of overweight/obesity in relation to dietary habits and lifestyle among 7–17 years old children and adolescents in Lithuania

**DOI:** 10.1186/s12889-015-2340-y

**Published:** 2015-10-01

**Authors:** Natalija Smetanina, Edita Albaviciute, Veslava Babinska, Lina Karinauskiene, Kerstin Albertsson-Wikland, Ausra Petrauskiene, Rasa Verkauskiene

**Affiliations:** Institute of Endocrinology, Medical Academy, Lithuanian University of Health Sciences, Eiveniu str. 2, LT-50009 Kaunas, Lithuania; Laboratory of Child and Youth Health, Institute of Health Research, Faculty of Public Health, Medical Academy, Lithuanian University of Health Sciences, Eiveniu str. 4, LT-50009 Kaunas, Lithuania; Faculty of Medicine, Medical Academy, Lithuanian University of Health Sciences, Mickeviciaus str. 9, LT-44307 Kaunas, Lithuania; Goteborg Pediatric Growth Research Centre, Sahlgrenska Academy, Goteborg University, SE-41685 Goteborg, Sweden

**Keywords:** Overweight, Obesity, Underweight, Children, Adolescents, Schoolchildren, Body mass index, Dietary habits, Physical activity

## Abstract

**Background:**

Until recently increasing prevalence of overweight and obesity among pediatric population in Europe and worldwide contributes to major well-known risks for metabolic consequences in later life. The aim of this study was to determine the prevalence of overweight/obesity among children and adolescents in Lithuania and assess its association with energy balance related behaviors as well as familial demographic and socioeconomic factors.

**Methods:**

Cross-sectional study included 3990 7–17 years old schoolchildren from 40 schools of Kaunas region, Lithuania. Study participants underwent anthropometric measurements. Body mass index (BMI) was evaluated according to International Obesity Task Force (IOTF) criteria for children and adolescents. Children and adolescents and their parents filled in the questionnaires on parental sociodemographic characteristics, dietary habits, TV watching time, and family socioeconomic status.

**Results:**

The prevalence of underweight, overweight, and obesity among boys and girls was 6.9 and 11.7 % (*P* < 0.05), 12.6 and 12.6 % (*P* > 0.05), and 4.9 and 3.4 % (*P* < 0.05), respectively. Obesity was significantly more prevalent in the 7–9 years old group (6.7 and 4.8 % in boys and girls, respectively, *P* < 0.05). Lower meals frequency and breakfast skipping were directly associated with overweight/obesity (*P* < 0.05); however, physical inactivity was not associated with higher BMI. Children‘s overweight/obesity was directly associated with lower paternal education and unemployment (OR 1.30, *P* = 0.013 and OR 1.56, *P* = 0.003, respectively).

**Conclusions:**

The prevalence of overweight and obesity among 7–17 years old Lithuanian children and adolescents was more prevalent in younger age, still being one of the lowest across the European countries. Meals frequency, breakfast skipping, paternal education and unemployment as well as a family history of arterial hypertension were found to be associated with children’s and adolescents’ overweight/obesity.

## Background

During the last 2 decades overweight and obesity have increased globally among children, adolescents, and adults [1]. The reviews of recent studies, however, showed an establishment and, in some countries, even a decrease in rates of obesity among children and adolescents [2, 3]. Despite these positive tendencies, obesity remains a major public health issue as it is well known to increases the risk of cardiovascular diseases, diabetes, hyperlipidemia, and musculoskeletal disorders in adults [4–6]. Recent studies have shown that obesity is less prominently associated with morbidity in adolescence but is a strong precursor for obesity and related morbidity in adulthood, with 50 to 80 % of obese adolescents becoming obese adults [6].

Previous studies reported a large variation in the prevalence of childhood overweight and obesity ranging from 2.9 to 44.4 % across various countries [7–14], with significant gender differences and considerable impact of familial socioeconomic background [15–17] highlighting the importance of familial psychosocial environment [18]. Genetic factors, absence of breastfeeding, inappropriate eating habits in the early years of life [19], consumption of large quantities of beverages rich in sugar, breakfast skipping, high-energy, high fat and low-fiber food [20], insufficient physical activity, and shortened night-time sleep duration [7, 17] were shown to be related with overweight and obesity in children and adolescents.

Studies addressing the issue of obesity among children and adolescents in Lithuania are scarce; to our knowledge, since 2002, no study investigating the prevalence of obesity among children has been carried out in Lithuania. World Health Organization (WHO) Childhood Obesity Surveillance Initiative (COSI) project presented data on the level of overweight among 6–9 year-old children [21]. Therefore, the aim of this study was to determine the prevalence of overweight and obesity among 7–17 years old children and adolescents of Kaunas region, Lithuania, and to assess its association with energy balance related behaviors as well as familial demographic and socioeconomic factors.

## Methods

This cross-sectional survey included 3990 7–17 years old schoolchildren from 40 schools of Kaunas region, which were randomly selected from the institutional registry list of the Ministry of Education and Science. The calculated population-based sample was 5000 schoolchildren; the response rate was 79.8 %. The study was conducted from October 2008 to February 2010. Intra- and inter- observer variability were 0.81 and 0.73, respectively, with the ranges 0.0-0.2 kg for weight and 0.0-0.4 cm for height. Agreement between observers Cohen’s kappa was 0.89. The study was performed in collaboration of Institute of Endocrinology with the Laboratory of Child and Youth Health of Health Research Institute of Lithuanian University of Health Sciences. Lithuanian Bioethics Committee approved the study. Written informed consent was obtained from the parents of all study participants.

The measurements were performed by the same trained and standardized researchers using the same standard tools. Inter- and intra-investigators validation process showed no significant variation. Height was measured to the nearest 0.1 cm with a portable SECA stadiometer (Seca®214). Weight was measured to the nearest 0.1 kg using portable SECA electronic scales (Seca®813). Body mass index (BMI) was calculated by using the standard equation: BMI = weight (kg)/height (m^2^).

The BMI category (underweight, overweight, and obesity) was defined using the International Obesity Task Force (IOTF) BMI cut-offs according to age and gender [22, 23].

Data of study participants’ dietary habits, physical inactivity and family socioeconomic status were collected from the study questionnaire, which was formed of modified WHO questionnaires (conducted by Health Behaviour in School-aged Children (HBSC) and COSI study groups) [24, 25]. The study questionnaire included 10 questions on food intake items (food frequency questionnaire (FFQ) part: breakfast eating frequency per week and meal frequency per day, consumption of different meals (fresh fruits and vegetables, sweets, sweetened beverages, energy-dense fast food) frequency per week), 4 questions on screen time (TV and personal computer (PC) use) on weekdays and weekends (how many hours do respondents spend daily) and 5 questions on family socioeconomic status (parental marital status (live in single or two parents’ family), parents’ education level, employment and type of their job).

All study children/adolescents were asked to fill-in the questionnaire: parents of younger age (7–9 years old) participants filled-in the questionnaire at home and older children and adolescents filled-in it themselves at school. Anthropometric measurements of study participants were done at school.

For further analysis, schoolchildren were grouped into 3 age groups: 1) 7 to 9 years old, 2) 10 to 13 years old, and 3) 14 to 17 years old. The participants were asked how many times per day they had their meal. The answers were combined into 3 groups: 1) “3 or fewer times per day”, 2) “4-5 times per day”, and 3) “more than 6 times per day”. The answers of the question “How often does your child have his/her breakfast” were combined into 3 groups as follows: 1) “Everyday” (“Everyday” and “4-6 times per week”), 2) “1–3 times per week”, and 3) “Never”. Physical activity was evaluated by questions about watching TV, time spent by the computer during working days and if did they spend more time with the same activity during weekend. The answers to the questions about TV and PC use were grouped as follows: 1) “never”, 2) “1–2 h per day”, and 3) “more than 3 h per day”.

Statistical analysis was performed using the SPSS 20 software package for Windows. The two-sided z test was used to assess differences between study groups. Logistic regression analysis was performed in order to identify factors significantly associated with overweight and obesity. A *P* value <0.05 was considered significant.

## Results

### Nutritional status

A total sample of 3990 children and adolescents aged 7–17 years were included in the study (1920 boys (48.1 %) and 2070 girls (51.9 %)).

Table 1 shows the distribution of study participants by age and gender. Study results showed that 9.4 % of children were underweight, 73.9 % had normal weight, 12.6 % were overweight, and 4.1 % were obese.

**Table 1:** Distribution of 7–17 years old children and adolescents by gender and age

Age group	Boys, number (percentage)	Girls, number (percentage)	Total, number (percentage)
7–9 years	807 (42.0)	829 (40.1)	1636 (41.0)
10–13 years	606 (31.6)	685 (33.1)	1291 (32.4)
14–17 years	507 (26.4)	556 (26.8)	1063 (26.6)
Total	1920 (100.0)	2070 (100.0)	3990 (100.0)

Distribution of nutritional status by genders is presented in Table 2. The percentages of underweight boys were significantly lower in all 3 age groups as compared with the corresponding percentages in girls, meanwhile overweight and obesity were significantly more prevalent among 14–17 years old and 7–9 years old boys, respectively, than among the girls of the same age groups (*P* < 0.05).

**Table 2:** Prevalence of underweight, normal weight, overweight and obesity by gender among 7–17 years old schoolchildren

	7-9 years		10-13 years		14-17 years	
	Boys	Girls	Boys	Girls	Boys	Girls
Underweight	5.7*^,^**	8.9***	6.9*	12.7	8.9*	14.6
Normal weight	74.5	71.8	76.9*	69.3	75.9	76.8
Overweight	13.1	14.5**	11.6	14.9**	12.8*	6.8
Obesity	6.7**	4.8**	4.6	3.1	2.4	1.8

Values are percentages

*P < 0.05 in comparison with girls

**P < 0.05 in comparison with 14–17-year age group

***P < 0.05 in comparison with 10–13-year and 14–17-year age groups

Comparison of schoolchildren by the BMI category and age within the gender groups showed that overweight was significantly more prevalent among the 7–9 and 10–13 years old girls than their older counterparts. The percentage of both obese boys and girls was significantly higher in the youngest than the oldest age group. Underweight was significantly less prevalent in the 7–9 year-old group in both boys and girls.

### Nutritional status & dietary habits

The analysis of dietary habits showed that 58.4 % of schoolchildren had their breakfast every day. The percentage of schoolchildren eating breakfast daily decreased with age: 66.5 % of 7–9 years old children had their breakfast daily, while this percentage among 10–13 year olds and 14–17 year olds was 57.9 and 52.3 %, respectively. Overweight and obese children and adolescents skipped breakfast more often than those of normal or low weight; however, this difference was not significant. Boys had breakfast daily significantly more often than girls (61.5 and 55.9 %, respectively; *P* < 0.05) (data not shown).

**Fig. 1:**
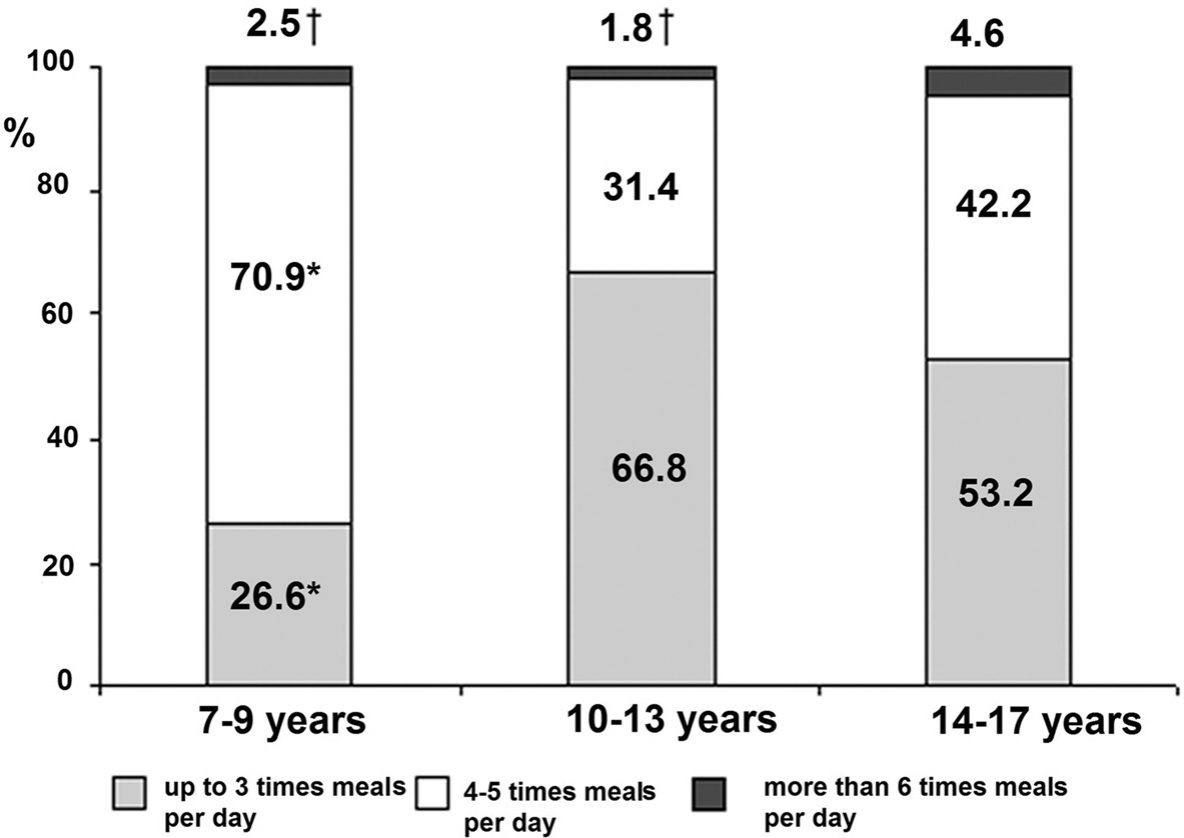
Distribution of meal frequency per day by the age groups. *P < 0.05 in comparison with 10–13-year and 14–17-year age groups. †*P* < 0.05 in comparison with 14–17-year age group

The majority (70.9 %) of 7–9 years old children had their meal 4–5 times per day, which was significantly more frequent compared to the 10–13 and 14–17 year-old groups. The greatest percentage of schoolchildren eating 6 and more times per day was among the oldest schoolchildren, significantly different from that of their younger counterparts (4.6 % versus 2.5 % and 1.8 %, *P* < 0.05). Less than half (46.7 %) of schoolchildren had their meals 4–5 times per day, and half (50.3 %) of schoolchildren ate 3 or fewer times per day, equally boys and girls. Figure 1 depicts the distribution of study participants by the age groups and number of meals per day. Less than one-third (26.6 %) of 7–9 years old schoolchildren had their meals 3 or fewer times per day, meanwhile the percentage of rarely eating children increased with age: in 10-13 and 14–17 year-old adolescents it was 66.8 and 53.2 %, respectively, with the difference being significant between age groups (*P* < 0.05).

**Fig. 2:**
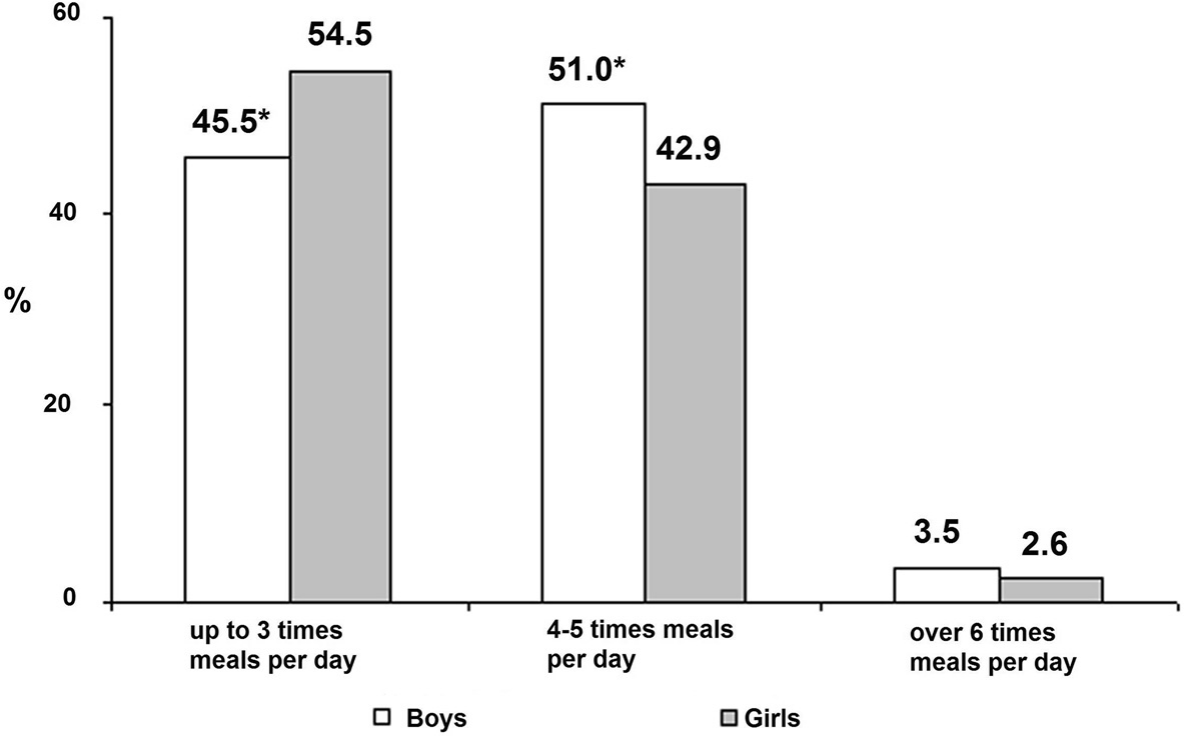
Distribution of meal frequency per day by gender. *P < 0.05 in comparison with girls

The comparison of meals per day by gender showed significant differences: girls had their meals significantly less times per day than boys (*P* < 0.05) (Fig. 2).

**Fig. 3:**
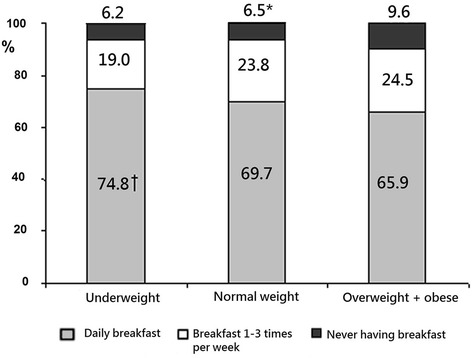
Distribution of breakfast eating rate per week by the BMI category. *P < 0.05 in comparison with overweight + obese group. †*P* < 0.05 in comparison with normal weight and overweight + obese groups

Figure 3 shows the distribution of schoolchildren by the BMI category and the frequency of breakfast eating. Underweight children and adolescents had their breakfast every day more frequently than those with normal weight or overweight/obese (74.8 % versus 69.7 % and 65.9 %, respectively; *P* < 0.05). Every tenth overweight or obese child/adolescent never had his/her breakfast, and overweight and obese children and adolescents were significantly more likely to skip breakfast than normal weight counterparts (9.6 % versus 6.5 %, *P* < 0.05).

Daily food preferences are presented in Table 3. Only every third 7–9 years old and every fourth 10–13 and 14–17 years old child consumed fresh fruits every day. Boys and girls aged 7 to 9 years consumed fresh fruits more frequently than those aged 14 to 17 years (*P* < 0.05). Both 7–9 years old boys and girls consumed fresh vegetables less frequently than their older counterparts did (*P* < 0.05). Boys aged 10 to 13 and 14 to 17 years ate candies and chocolate less frequently than the girls of the same age groups did, but reported eating snacks more frequently than girls did. Lemonade/soft drinks consumption was more frequent among 10–13 and 14–17 years old children and adolescents compared to the younger group (*P* < 0.05).

**Table 3:** Comparison of frequencies (%) of daily (more than 4 times per week) consumed meal type and soft drinks between different age groups, adjusted for age and gender

Food/drink	7-9 years		10-13 years		14-17 years	
	Boys	Girls	Boys	Girls	Boys	Girls
Fresh fruits	33.0**	31.1**	27.4	28.3	23.8	24.0
Fresh vegetables	18.3***	16.1***	25.4	26.3	25.3	29.2
Candy/chocolate	13.7	12.1***	16.9*	22.8	14.4*	22.4
Biscuits/cake	8.5	5.7	5.7	3.4	6.0	5.2
Lemonade/soft drinks	6.0***	4.8***	14.7	12.6	12.5	10.8
Snacks (chips, peanuts, etc.)	2.7**	2.0	7.6*	2.4	5.2*	1.6
Fast food	1.4***	1.5****	4.9	3.5	4.2*	2.0

Values are percentages

*P < 0.05 in comparison with girls

**P < 0.05 in comparison with 14–17-year-age group

***P < 0.05 in comparison with 10–13-year and 14–17-year-age groups

****P < 0.05 in comparison with 10–13-year-age group

### Nutritional status & physical inactivity

**Fig. 4:**
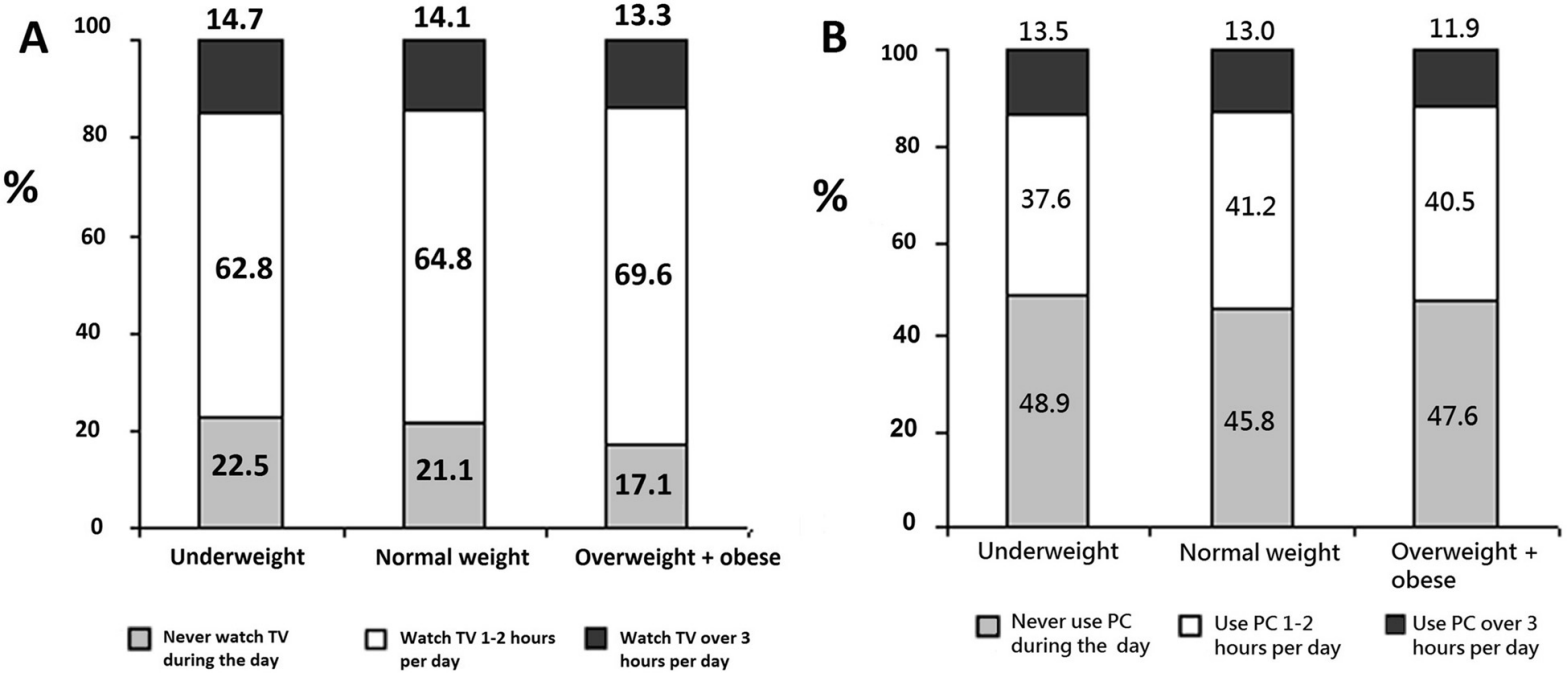
Distribution of time spent watching TV (**a**) and at the computer (**b**) during the day between schoolchildren by the BMI category

Nearly half of children and adolescents did not spend any time at the computer during the week or spent only 1–2 h per day (46.4 and 40.7 %, respectively). Only 12.9 % of the respondents spent 3 h per day and more by the computer. Nearly two-thirds (65.4 %) of the schoolchildren watched TV 1–2 h during the day, 14 % watched TV for 3 h and longer, and every fifth schoolchild (20.6 %) never watched TV or watched it only 30 min per day. Older schoolchildren spent more time watching TV and by the computer than their younger counterparts: in the 7–9 years old group 10.8 % spent 3 and more hours watching TV and 2.6 % spent 3 and more hours at the computer, compared with 16.2 and 17.3 % in the 10–13 years old group, respectively, (*P* < 0.001). However, the comparison of schoolchildren by the BMI category and time spent at the computer or watching TV showed no significant differences (Fig. 4).

### Nutritional status & socio-demographics

Table 4 summarizes the results of logistic regression analyses; comparisons of family composition and frequency of familial hypertension/diabetes between the underweight/normal weight/overweight groups were made adjusted to parental educations status.

**Table 4:** Distribution of parental socio-demographic characteristics according to the children/adolescents’ nutritional status

Factors	Underweight (%)	Normal weight (%)	Overweight, including obesity (%)
Maternal education			
Secondary	9.5	73.0	17.5
Higher Non-University	9.5	75.1	15.4
Higher University	9.4	75.0	15.6
	*P* = 0.760		
Paternal education			
Secondary	10.1	73.1	16.8*
Higher Non-University	8.5	79.6	11.9
Higher University	9.9	74.6	15.5
	*P* = 0.201		
Family composition^a^			
Living with both parents	10.6	72.7	16.7
Living with a single parent	9.2	75.5	15.3
	*P* = 0.397		
Family history of arterial hypertension^a^			
No	10.0	75.5	14.5
Yes	10.1	71.9	18.0**
	*P* = 0.042		
Family history of diabetes^a^			
No	10.0	73.8	16.3
Yes	8.6	73.3	18.2
	*P* = 0.348		

*P < 0.05, z test comparing with Higher Non-University education

**P < 0.05, z test comparing with no family history of arterial hypertension

^a^Adjusted to parental educational status

Having a family history of arterial hypertension was associated with child/adolescent’s overweight and obesity (OR 1.47, 95 % CI 1.31-1.66, *P* < 0.001). Moreover, paternal unemployment was associated with child/adolescent’s obesity (OR 1.79, 95 % CI 1.40-2.28, *P* < 0.001), meanwhile maternal unemployment as well as family history of diabetes had no impact on child/adolescent’s weight (OR 0.97, 95 % CI 0.88-1.06, *P* = 0.47 and OR 1.46, 95 % CI 0.91-1.45, *P* = 0.25, respectively). Living in an incomplete family was not associated with child/adolescent’s overweight/obesity (OR 0.902, 95 % CI .70-1.16, *P* = 0.43). Parental education was not associated with children overweight/obesity (*P* > 0.05), except fathers with higher non-university education, whose children/adolescents were nearly 1.5 times less likely to be overweight or obese than schoolchildren whose fathers had primary education (OR 0.67, 95 % CI 0.46-0.97, *P* = 0.03) (Table 4).

## Discussion

### Prevalence of overweight/obesity

The prevalence of overweight is high among school-aged children across Europe, with an especially worrisome prevalence in South European countries (such as Italy, Spain, and Greece) [7]. On the other hand, the US Centers for Disease Control and Prevention (CDC) as well as some European centers reported the prevalence of overweight and obesity plateauing and even decreasing [26].

This study showed that the prevalence of underweight, overweight, and obesity among 7–17 years old children and adolescents was 9.4, 12.6, and 4.1 %, respectively, with a higher prevalence among boys. These results are in line with the results of other studies reporting higher rates of overweight or obesity in boys than girls [10, 27, 28]. As compared with the data published in other countries, Lithuania still has the lowest prevalence of overweight and obesity among 7–17 years old children and adolescents in Europe, being similar to that in Poland and Latvia [29, 30].

A Lithuanian study by Tutkuviene showed that in 2000–2002, the prevalence of overweight varied from 1.5 to 10.5 % in girls and 2.6 to 9.9 % in boys, respectively, and the prevalence of obesity ranged from 0 to 2.9 % in girls and 0 to 4.37 % in boys, respectively [31]. In contrary to study by Tutkuviene, in our study a higher prevalence of overweight in both sexes and higher prevalence of obesity only among girls was found. In our study, the prevalence of overweight and obesity was lower among adolescents compared with younger age school children. Similar tendency was reported in the studies by Pizarro et al. and Lazzeri et al. [28, 32]. Since the cross-sectional design of our study do not allow drawing conclusions on the reasons for these differences, we may only hypothesize that older children and adolescents become more interested in their physical appearance, especially girls, they have less structured life and perhaps have more outdoor activities with their counterparts compared to younger children.

### Dietary habits

Inappropriate eating habits in the early years of life can lead to permanent disorders with serious consequences in adulthood [19]. Eating behaviors such as overeating, unhealthy food, breakfast skipping, and rare participation in family meals have been reported to be associated with the risk for being overweight among preadolescents [7, 33]. Several studies have shown that obese children tend to skip meals, especially breakfast [7], more often than non-obese children [34–39], and this habit strengthens with age [36]. The results of this study showed that one-third of Lithuanian children and adolescents skipped their breakfast daily, girls even more frequently than boys. Having in mind that breakfast skipping is associated with obesity, this observation does not explain a lower prevalence of overweight and obesity among girls, but may highlight a problem of under-nutrition among girls as seen in this study, since underweight was more common among girls than boys and its prevalence increased with age.

Our results showed that only half of schoolchildren had their meals during the day as recommended (4–5 times); other half of children and adolescents ate irregularly (3 or fewer times per day), especially girls. Daily eating frequency has been reported to be inversely associated with overweight [40–42]: eating 3 or fewer meals per day or inadequate intake of carbohydrates [43] has been shown to be associated with a greater likelihood of being overweight or obese.

The present study found that 7–9 years old children of both genders consume fresh fruits more than 10–17 years old adolescents, but vegetables consumption is higher among adolescents compared to younger children. According to cross-national Health Behavior in School-aged Children (HBSC) study conducted in 2002, 2006 and 2010, in Lithuania school-aged children have low intakes of fruits and vegetables. Only 21.1 % of boys and 27.1 % of girls reported daily fruit consumption. Similarly, 24.9 % of boys and 29.6 % of girls disclosed vegetable intake at least once daily [44]. On the contrary, the consumption of fast food with sweetened beverages was considerable, especially among girls. Therefore, possible compensation for insufficient nutrition with unhealthy food is an important public health issue in these age groups, highlighting the need for elaborating comprehensive educational strategies on healthy nutrition in schools programs.

As published earlier, children with normal weight eat vegetables, fruits and berries more frequently than overweight children [45], and the consumption of vegetables, cooked meals and eating dinner are negatively associated with overweight in children [46]. However, some studies have reported that obese adolescents are more likely to be advised a healthy diet than adolescents who have normal weight [47]. As shown in this study, overweight and obese children and adolescents reported to eat healthier and to consume sweetened beverages less frequently, but skipped breakfast more often than non-obese children/adolescents. This finding might explain a weak dietary influence on the etiology of obesity in some subjects. Alternatively, it may be due to social desirability and underreporting bias by overweight/obese respondents.

### TV watching and computer use

Sedentary behavior has been shown to be associated with higher BMI, weight gain and adiposity in children and adolescents [48]. Greater TV, computer, video game and other media exposure time in children is increasing and is associated with adverse health outcomes such as becoming overweight [49]. A range of indicators of sedentary lifestyle, such as TV watching, especially during the meals, and the presence of a TV in the child’s bedroom, has been reported to be associated with overweight and obesity [35, 40, 50–52].

Time spent at the computer or TV during the week in this study was used as a proxy for physical inactivity, but there was no association between time spent at the computer or TV and being overweight/obese. In our study, nearly two-thirds of schoolchildren spent up to 2 h at the computer/TV, as it advised by healthy lifestyle recommendations. However, there was a trend toward longer TV watching and computer using time among older children and adolescents, probably due to diminished parental control. Such inactivity might contribute to the increasing prevalence of overweight among boys.

Absence of the association between the overweight/obesity and TV/computer use do not reflect the other side of overweight/obese children inactivity: these children might have passive hobbies (music, arts, chorus, etc.) contrary to normal weight/underweight counterparts who could be involved in more active hobbies such as basketball and football, athletics, tennis, swimming, street dance, etc. Previously published that overweight/obese children are more sedentary and report more screen time than normal weight children [53]. This notion is supported by earlier report of Nitzan Kaluski et al., who did not find any relationship between time spent at the computer, viewing TV or videos, or listening to music as a measure of physical inactivity and obesity [54]. Another explanation could be a direct type of the question in the questionnaire (TV/computer use), which doesn’t cover other gadgets use (PlayStation, Smartphone browsing, tablets, and consoles).

As suggested by some authors, a negative effect of TV/computer use may be associated with increased intake of sweets and snacks, both due to an influence on changing eating behavior and to greater exposure to advertisements of food high in sugar and fat [36, 52]. However, the study by Carson and Janssen reported no relationship of BMI with snacking while watching TV and junk food consumption [50, 55] . Therefore, the impact of snacking during watching TV on weight gain needs further elucidation.

### Socio-demographic environment

Family socioeconomic status has been reported to be inversely related to the prevalence of overweight and obesity [28] with the highest prevalence of overweight being in the lowest socioeconomic groups [8, 36]. Previous studies showed that higher level of maternal or paternal education were associated with lower risk of being overweight in children. In our study paternal education and unemployment were associated with child/adolescent’s overweight and obesity. Recently published Finnish LATE study found that paternal education had inverse association mediated by parent’s own BMI with overweight in older boys. Direct association between parental education and childhood overweight was stronger than indirect association. However, maternal education had only indirect association [56]. Parental education is a strong socioeconomic gradient related to eating behaviors and television viewing time [57]; this factor was also found to be positively associated with the prevalence of optimal level of physical activity [54]. Children of less educated parents with low-income consume nutrient-poor but energy-dense beverages more frequently instead of nutrient-dense beverages [7, 58]. However, our study found no significant associations between family composition, maternal education, and maternal employment status and child/adolescent’s BMI. In addition, in other studies family composition had an impact on children’s BMI: children/adolescents in single-parent household watch more TV, eat more food high in fat and sugar and less fresh fruit and vegetables than children from dual-parent households, although not found in our study.

### Strengths and limitations of the study

The present study has several strengths. First, study represents Kaunas region, which is central and nationally homogenous part of the country, second largest city of Lithuania. The study was performed by the same trained research team using the same standard tools. Study cohort is a representative sample of the whole country having in mind small population of Lithuania.

This study also has few limitations. Firstly, cross-sectional study design does not allow disclosing trends of change in adiposity status and energy balance related behaviors in the same children over time. Secondly, we could not avoid questionnaire fill in bias as overweight/obese respondents might consciously conceal improper dietary habits and physical inactivity. Finally, the time of physical activity on weekends was not specifically highlighted by the questionnaire.

## Conclusions

Our study showed that the prevalence of overweight and obesity among 7–17 years old Lithuanian children and adolescents is still one of the lowest across European countries. Eating behaviors and breakfast skipping was related with higher obesity level; however, physical inactivity was not associated with overweight and obesity in children and adolescents in this study. Lower paternal education and unemployment were found to be important obesogenic factors in children and adolescents. Results of our study have important implication in planning public health strategies, highlighting the need for elaborating comprehensive educational programs on healthy nutrition in schools, since energy balance related behaviors in youths is a modifiable risk factor for obesity and later metabolic complications.
